# Easy Read… Easy English… Plain Language? Decision‐Making in the Production of ‘Easy’ Information in Australia

**DOI:** 10.1111/jar.70021

**Published:** 2025-02-17

**Authors:** Ariella Meltzer, Emma Barnes, Ayah Wehbe

**Affiliations:** ^1^ Centre for Social Impact University of New South Wales Sydney Australia; ^2^ Arts, Design and Architecture University of New South Wales Sydney Australia

**Keywords:** accessible information, Easy English, Easy Read, intellectual disability, low literacy, Plain Language

## Abstract

**Background:**

In Australia, several formats of written information are made for people with intellectual disability and/or low literacy, such as Easy Read, Easy English and Plain Language. More understanding is however required about the decision‐making behind their features, as it is not always clear which formats use which features or for what reasons.

**Method:**

Twelve semi‐structured interviews were conducted with leaders/senior staff in Australian accessible information provider organisations who make ‘easy’ information. Data were thematically‐analysed, via a two‐stage deductive‐inductive process.

**Results:**

The results show overlaps between different ‘easy’ information formats in Australia. ‘Easy’ information provider organisations make choices about what reading‐level and images to use based *how* they see their envisioned audience *using* the information and *what* will make their envisioned audience feel *recognised/empowered*.

**Conclusions:**

No single ‘easy’ format will suit everyone. ‘Easy’ information providers need to be more specific about the audience and associated features of their products.

## 1 Introduction

The right of people with disability to accessible information is recognised in Article 21 of the United Nations Convention on the Rights of Persons with Disabilities. Accordingly, accessible information is increasingly recognised as a key element of inclusive practice, and governments, service providers, businesses and community groups are increasingly providing information in formats for people with a range of types of disability.

For people with intellectual disability and other disabilities affecting cognition/literacy, formats with simple phrasing and other comprehension supports are often produced. Such materials are variously titled Easy Read, Easy‐to‐Read, Easy English, Plain Language and/or Plain English (henceforth collectively called ‘easy’ information). Table 1 summarises their features, indicating both similarities and significant variation in how some features are applied.

**TABLE 1:** Features of ‘easy’ information formats.

Formats	Common features	Description
Easy Read Easy‐to‐Read Easy English	Simple words. Short, clear sentences. Reduced punctuation. Dot points. Large print. Extra white space on page. Pictures to support text. Sometimes explains hard words.	Formats termed Easy Read, Easy‐to‐Read and Easy English have simple words, short/clear sentences, large‐print text, extra white space on the page and pictures to support comprehension (Centre for Inclusive Design 2020; Inclusion Australia 2023). There is much variation in exactly how simple the phrasing is (i.e., what reading‐level^a^ is used); whether photos or graphics are used; and whether ‘hard words’ are avoided or explained (Basterfield 2020). Some authors state that Easy Read and Easy English can be consistently distinguished by which features they have, with Easy English being considered ‘easier’ than Easy Read and commonly having symbols rather than Easy Read's photos (Basterfield 2020). However, this delineation is not consistently used by everyone. The audience of these formats also varies, at times including people with intellectual disability, other disabilities or circumstances leading to low literacy, and people who use English as a second language.
Plain Language Plain English	Everyday words. No jargon. Clear, active sentences. Short paragraphs.	Plain Language and Plain English documents use everyday words, no jargon and active tense, but their phrasing is not as simple as Easy Read, Easy‐to‐Read or Easy English (Centre for Inclusive Design 2020). They do not use pictures to assist comprehension (Centre for Inclusive Design 2020). Their audience is typically the whole population, but with attention to those who find complex/dense text difficult.

^a^ Reading‐levels are standardised measures, usually aligned with a Grade 1–12 education system, of how hard text is to read.

What is not immediately evident from Table 1 is exactly how contested and political ‘easy’ information formats can be. While ‘easy’ information is increasingly seen as a tool of inclusion and embraced by governments/not‐for‐profits/private businesses as a way of implementing human rights for people with intellectual disability, there remains much discussion about the evidence‐base behind the formats.

Much research has questioned whether ‘easy’ information formats effectively deliver their message (Hurtado, Jones, and Burniston 2014; Sutherland and Isherwood 2016; Chinn and Homeyard 2017; Al‐Shirah 2023). In particular, research has queried the efficacy of pictures for comprehension support (Jones, Long, and Finlay 2007; Poncelas and Murphy 2007) and some has questioned the language choices made (Buell et al. 2024). Studies have also taken place about the support required to use ‘easy’ information (Mander 2016; Chinn 2019a), and about market forces which promote the production of ‘easy’ information, but not necessarily in ways led or even always co‐produced by people with intellectual disability (Chinn 2019b).

Further, some jurisdictions are ahead of others in guiding the production of ‘easy’ information. For instance, the United Kingdom (UK) and Europe guide these formats through relatively high‐profile national/continent‐level tools such as the UK Association for Accessible Formats' guide on Easy Read documents and the European Easy‐to‐Read Standards. Such tools provide some guidance, and hopefully agreement, on how (at least some of) the formats are to be crafted/implemented. Yet, other countries—such as Australia—very commonly use ‘easy’ information formats, but do not have agreed national‐level guidance on their features/implementation. The closest is perhaps the new Easy Read guidance in the online edition of the Australian Government Style Manual, yet this is intended primarily for government‐use rather than the broader community, and only differentiates between Easy Read and Plain Language, not other related formats. The net effect is that in some jurisdictions the use of these formats is even more unclear than others.

Even in the jurisdictions that are relatively advanced in guiding ‘easy’ information formats, there are important impacts of the contested context in which these formats exist. The lingering questions about evidence‐base combined with the market forces and political pressure to keep creating such materials perhaps prompts organisations who make ‘easy’ information to vouch strongly for their own materials. Writing from the UK, Chinn (2019b) notes that there is very little consensus among ‘easy’ information providers about how such materials should be produced, a situation also reflected in Table 1 above and compounded, as Chinn notes, by ‘easy’ information end‐users often being treated as a homogenous group with homogenous needs, when this is not actually the case. In this context, Chinn (2019b) finds that many providers of ‘easy’ information end up producing materials that meet the preferred design elements of their own ‘local audiences’, but do not necessarily test these or get feedback beyond their local audience. Chinn finds that this creates a series of ‘imagined contexts’ for the reception of ‘easy’ information, ones which do not necessarily cover the full picture of its possible uses.

Set against this background, this paper aims to understand more about the situation of ‘easy’ information in Australia. As noted earlier, ‘easy’ information formats are commonly produced in Australia. Yet, as a country without clear national guidance on the features/implementation of such formats, Australia is perhaps even more susceptible to some of the challenges stemming from the contested evidence‐base for ‘easy’ information and from the potential for Chinn's (2019b) issue of ‘local audiences’. As such, this paper seeks to answer the research questions: *What feature decisions do Australian* ‘*easy*’ *information providers make when crafting their products*? *How much do their decisions vary as a sector*, *and what rationales are provided? What is the impact for the collective* ‘*easy*’ *information sector in Australia*?

## 2 Method

This paper draws from a qualitative study of Australian accessible information provider organisations (Meltzer et al. 2024a). The study was about creation of accessible crisis information for use during the pandemic, bushfires and floods that have affected Australia in recent years. The study included those who make a range of formats, including ‘easy’ information and also formats such as Australian Sign Language, captioning, screen‐readers, audio and Braille. However, the high rate of representation of ‘easy’ information organisations allowed the sub‐focus on ‘easy’ information production reported here.

The study received ethics approval from the University of New South Wales' Business School's Human Research Ethics Advisory Panel (HC230201). All participants/organisations gave written informed consent prior to their interview.

Of note, the study sits alongside an inclusive research approach—while not collecting data from end‐users with intellectual disability, it illustrates the perspectives of those who closely listen to and serve their accessibility requirements.

### 2.1 Research Design and Data Collection

The study was exploratory. Semi‐structured in‐depth interviews were undertaken with senior representatives of accessible information provider organisations. The organisations were recruited through an invite sent to their organisational email address and then an opt‐in process. Organisations were eligible to participate if they create/provide accessible information to people with disability in Australia. An invite list was compiled via an online scan of relevant organisations, together with snowballing. While this recruitment method may have gaps, the invites were issued widely, including to organisations in multiple Australian states/territories.

In‐depth interviewing was used, as appropriate for exploratory research where new insights are generated from scratch (Jain 2021). The questions covered:

- What ‘information accessibility’ means in concept and practice;
- How providers determine product features;
- Experiences of making accessible information;
- Facilitators/barriers to making accessible information;
- Resourcing/policy changes to assist accessible information production.

Interviews were 40–60 minutes, audio‐recorded and transcribed verbatim. Identifying details (e.g., individual/organisational names) were replaced with descriptors. The transcripts were coded thematically using NVivo 12 (QSR/Lumivero), as described below.

### 2.2 Data Analysis and Researcher Positionality/Reflexivity

**FIGURE 1:**
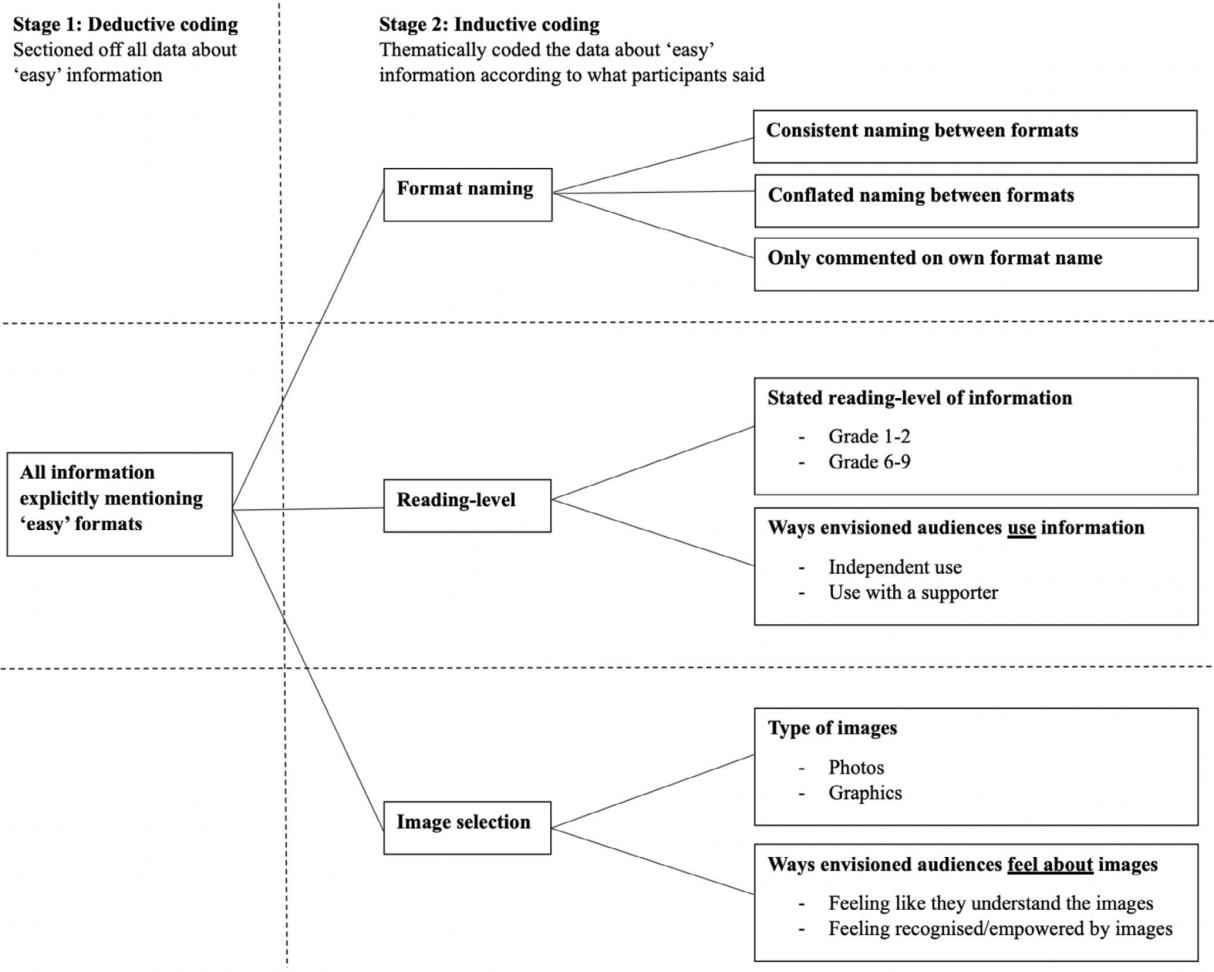
Summary of themes.

Analysis from scratch was chosen to ensure the findings were specific to the Australian context. The data were first deductively‐coded according to the focuses/topic‐elements listed above (by EB), to section the data according to different elements of the overarching research focus. This included sectioning off all information about ‘easy’ information. Each section was then inductively‐coded (by AM) to reflect the themes in what participants said, broadly applying Braun and Clarke's (2006) thematic analysis process. Data in the final themes were checked by two authors (EB, AW) for accuracy/representativeness to the original dataset. Themes related to ‘easy’ information are presented in this paper. As displayed in Figure 1, they cover format naming, reading‐level and image selection, and the broader commentary/implications of these areas. Other publications include findings in areas beyond ‘easy’ information (Meltzer et al. 2024a, 2024b).

It is important to acknowledge researcher positionality/reflexivity during analysis. Of the three authors, only one (AM) had prior substantial connection to ‘easy’ information. Prior to the research, EB was not connected to accessible information and, while AW has written ‘easy’ information in her research work, her primary expertise in this study was leading other parts of the work focused on Australian Sign Language.

AM has written ‘easy’ information for research purposes for almost 15 years. She has completed ‘easy’ information training using both Grade 1–2 and 6–9 reading‐levels, as well as both symbols and pictures. While using predominantly Grade 6–9 and a mix of symbols/photos in documents she writes herself, the author has on occasion commissioned others to make Grade 1–2 information with symbols for her research dissemination.

Regarding reflexivity, the author purposefully aimed to listen to multiple ‘easy’ information providers, with attention to the rationale given by each for the features they use. Of note, she aimed to suspend assumptions about format names that should be applied to specific combinations of ‘easy’ information features, focusing instead on document content. While this may be considered a bias, it was intended to allow greater attention to the content/rationale mentioned by participants, without focusing on format‐naming debates.

### 2.3 Participant Organisations

As noted earlier, the study comprised interviews with senior representatives of organisations who make accessible information. Senior representatives were either organisational leaders or another person with senior responsibility for accessible information.

Seventeen organisations participated in the overall study. Most had a single staff member participate, except for two organisations who did group interviews (with two and four staff respectively). Of the 17 organisations, 12 made ‘easy’ information and are included in this paper. As shown in Table 2, six of these organisations were dedicated solely to making ‘easy’ information and the further six included ‘easy’ information while making other formats as well. The remaining five of the overall 17 organisations specialised solely in other formats (e.g., Australian Sign Language, captioning, screen‐readers, audio, Braille) and are not included in this paper. The organisations making ‘easy’ information came from a range of disciplinary backgrounds, including speech pathology, communications and disability advocacy.

**TABLE 2:** Sample by accessible format type.

Organisations focused solely on ‘easy’ information	*n* = 6
Organisations **including** ‘easy’ information **and** other formats	*n* = 6
Organisations focused solely on other formats	*n* = 5

## 3 Findings

The following sections detail the findings, discussing three areas: (1) lack of clarity in ‘easy’ information features and format naming; (2) variation in reading‐level and implications for understanding the purpose of or ways of using the ‘easy’ information; and (3) variation in images and implications for understanding the preferences of ‘easy’ information audiences.

### 3.1 Lack of Clarity in ‘Easy’ Information Features and Format Naming

One key finding was lack of clarity and definition in ‘easy’ information features and format naming. This finding confirms the definitional debates indicated earlier in this paper.

Some organisations saw Easy Read and Easy English as two different formats, consistently distinguished by their different choices on reading‐level and images. These participants saw Easy English as ‘easier’ than Easy Read:

So for me, Easy English is the simplest version… It uses less words, less sentences, the images are often simpler, but also not photos, so using little sketches of people… Easy Read is kind of that step between Easy English and Plain Language. So you can kind of use a range of images, you can include some slightly more complex ideas, it does have longer sentences, a longer document, less white space on the page (ORG_08).

The same distinction was however not made by everyone. Many other organisations either made documents titled Easy English that were closer to the features described by the participants above as Easy Read or vice versa. Alternatively, many organisations saw Easy Read and Easy English as interchangeable terms:

Easy English and Easy Read basically mean the same thing to most people that we work with (ORG_11).

We just have used [the term] Easy English. That's just the way that we say it. We know it's the same thing [as] Easy Read (ORG_03).

Further, some organisations also flagged confusion between what is Easy Read and what is Plain Language/Plain English. The quote below demonstrates this, with the additional confounding element that the reading‐level described below as Easy Read would be described by others as Easy English:

Easy Read is clear, concise, information that is presented in simple terms… around a Grade 1‐2 level… with a picture alongside it that matches the words… it's not Easy Read if the information there is actually quite difficult to understand. Like a lot of things that are called Easy Read, the words included in them are more like Grade 4‐7… I wouldn't call that Easy Read, I would call that a mix between Plain Language and Easy Read (ORG_11).

The effect of these different views is that it is very difficult in Australian ‘easy’ information production to understand the formats involved—a situation recognised by some participants:

… having [different] categories of something when they're both called ‘easy’… it's obviously very confusing… I think it's further confused by the fact that we don't have accessible information guidelines in Australia, so we don't have firm definitions around [the different formats] (ORG_11).

Overall, while some participants see clear differences between formats, others are either not clear on the differences or do not believe there are differences. This contributes to confusion between what is Easy English, Easy Read and Plain Language, and, perhaps, to whether there even are differences at all.

### 3.2 Variation in Reading‐Level and Implications for Understanding the Purpose of or Ways of Using ‘Easy’ Information

When ‘easy’ information provider organisations spoke about the reading‐level of their products, there was a lot of variation, with organisations citing anything from Grade 1–2 to Grade 6–9 reading‐levels. As noted earlier, some participants equated the Grade 1–2 reading‐level with Easy English, but others noted this as a feature of Easy Read. There tended to be more agreement that Plain English/Plain Language used a higher reading‐level. Beyond trying to identify a particular reading‐level with a particular format name—which cannot be done consistently for all study participants—some other insights are also important, related to what the reading‐level means about the envisioned purpose or ways of using the information. In many ways, these findings echo Chinn's (2019b) insight that there are a series of “imagined contexts” for the reception of ‘easy’ information.

A small number of organisations used language, phrasing and structuring at Grade 1–2 reading‐level. Some of these organisations focused on maximising the amount of text that readers could understand and use independently:

As far as practical, we're trying to get it so that it can be used directly by the intended audience without the need for that support person (ORG_13).

Others recognised that supporters would sometimes be there to help the reader, but focused on the ‘easy’ text (at Grade 1–2 level) being pitched/structured so that supporters were clarifying information verbatim, rather than needing to re‐interpret/explain it:

When I look at something that we create in Easy English, I know that a support worker is more likely to read and understand it, to be able to then have a more meaningful conversation, because the cognitive load has been removed. And so [the person with intellectual disability/low literacy] can now go, ‘Yep, I absolutely know what that means. I don't have any questions about what all those words [on the page] are that aren't [the ones] that you're trying to tell me’ (ORG_18).

In comparison, a more substantial number of organisations created documents at a Grade 6–9 reading‐level. These organisations noted that people who use their ‘easy’ information rarely read it completely independently, no matter the reading‐level, and the information is instead usually used with a supporter present, who may have a substantial role in helping the person access/understand it:

Easy English is not always something that's a stand‐alone document. For many people, it's a tool to a conversation. It's a document that you sit side‐by‐side with someone and have it explained to you or go through it (ORG_04).

Easy Read‐Easy English is designed to be read with a support person. So you might [have a]… service agreement… The support person can read through it with them, they can get an idea of what they're actually going to be signing. And if there's any questions… the support person can go to the complex version and help them to understand that a bit better (ORG_01).

This difference in how the purpose or ways of using ‘easy’ information are envisioned has implications for how the information is constructed. The provider organisations that focus on independent use/verbatim understanding of the text tend to be those who use the lower Grade 1–2 reading‐level. In comparison, those who envision that a supporter will almost always be present with a significant role in helping the person understand the text instead more commonly use the Grade 6–9 level. As one participant articulated, this allows the text in combination with the supporter to have a role in expanding the concepts, ideas and vocabulary that the person using the information is exposed to:

Easy English is always supposed to be read with a support person so that you can ask that person questions about the text and clarify meaning… you can read through an Easy English document, learn more about a concept or an idea, expand your vocabulary (ORG_15).

Understanding that exposure to new concepts, ideas and vocabulary is part of the purpose of some ‘easy’ information helps to explain why some ‘easy’ materials are more complex than others—because they need to have some somewhat more complex content to include the new concepts, ideas and vocabulary to be exposed to. In comparison, if the focus is instead on independent use/verbatim understanding of information then the content needs to be much more simply phrased, to enable independent reading and verbatim understanding by those with low literacy.

### 3.3 Variation in Images and Implications for Understanding the Preferences of ‘Easy’ Information Audiences

The other area where ‘easy’ information provider organisations vary is in the images they use. Some use photos (e.g., Photosymbols, stock photos) while others use graphics (e.g., general graphics, Compic/Boardmaker symbols). There are contradictions between the reasons different organisations cite for their respective image choices. The contradictions reveal much about variety in the preferences of the audiences of their respective ‘easy’ information products, exemplifying Chinn's (2019b) point that ‘easy’ information users are not a homogenous group with homogenous needs.

One contested issue is which kind of images are easiest to understand. Some organisations use Photosymbols, a UK‐designed photoset designed specifically for ‘easy’ information, which features people with disability, including many with intellectual disability. These organisations note that Photosymbols are preferred by the people with intellectual disability they work with, because the images look ‘real’ and include people with intellectual disability, which makes them easy for this group to relate to and interpret. They noted that some of the symbolic imagery in other graphics was hard for people with intellectual disability to understand:

[The members we surveyed] absolutely did not like cartoon Compic… They really didn't understand [it] … Things like a speech bubble: what is this? (ORG_04).

Line‐drawings or illustrations can actually be a little bit more difficult for people to interpret than, say, a colour photo that's been taken very simplistically and follows its own set of conventions (ORG_13).

However, other organisations who use graphics disputed these perspectives—instead highlighting the problems with photos and benefits of graphics. The limited generalisability of photos was an issue for this group, as was the cluttered nature of many stock photos:

Symbols allow people to generalize the concept as well, verses photos. Photos are very specific. So if I have a photo of a house, it's a photo of *that* house. But a symbol of a house is symbolic of all houses, which is quite important (ORG_15).

Photos can be quite cluttered in the background, and a bit hard for people with vision impairment to see. If they're not selected carefully, they can sort of just add confusion (ORG_01).

In comparison, they highlighted the benefits of graphics, especially picture sets that could be intentionally customised to maximise the reader's understanding:

You can pick key characters. So it might be that the main character in this Easy English document is a woman wearing a blue shirt. As you go through that document, you can keep that consistent to support the reader to rely on that's the main character (ORG_15).

A second issue was how the picture selection made the reader feel. Organisations making ‘easy’ information primarily for people with intellectual disability noted that photos are preferred by this group because they feel age‐appropriate, empowering and not infantilising, especially if people with intellectual disability are pictured doing empowering activities:

They thought [Compic pictures] were too childlike (ORG_04).

We have heard lots of times, if we give something that's got mostly graphics, ‘I'm not a child’. Those sorts of comments, and to us, as an advocacy organisation, we want people to feel empowered (ORG_11).

In comparison, those making information specifically for people who use alternative and augmentative communication (AAC) recognised that the use of graphics—specifically Compic/Boardmaker symbols that commonly appear in AAC systems—is more empowering for this group, as it is validating for them to see the same symbols that feature in their communication system in other documents/community settings:

I'm seeing you one‐to‐one in the office here in the clinic setting [working with a Compic/Boardmaker‐based communication system]. But when you go out into the community, you're seeing your language available to you as well… we put a lot of thought into what words we use on all of our communication tools, what symbols we use. Are they in line with what appear on a person's communication system? (ORG_15).

The other group preferring symbols were those making ‘easy’ information for people who might not identify as having a disability (e.g., people with low literacy for reasons other than intellectual disability). This was in part because symbols were understood to be more easily understandable for this group, but also because this group—who do not necessarily have a visible disability and may not even identify as having a disability at all—may not identify with disability‐focused imagery. Disability‐focused imagery was therefore not necessarily empowering for them.

These debates reveal much about how the image selection decisions of ‘easy’ information provider organisations reflect the preferences of their audiences. Set against a situation where there is much debate about the effectiveness of different image choices (Jones, Long, and Finlay 2007; Poncelas and Murphy 2007), how the images make the audience *feel* comes to the fore. Different types of images speak to different audiences and make the ‘easy’ information *feel* appropriate and empowering for them—but in different ways for different groups. This means, however, that no single type of image will ever suit all audiences.

The finding that no single type of image will suit everyone is important because, as Chinn (2019b) has also noted, there was a tendency among many of the participants in this study to assume that one ‘easy’ format type could suit multiple audiences:

‘Who is it for?’ Again, I think we go back to that piece about as many people as possible is who it's for (ORG_15).

Easy Read information is not just for people with an intellectual disability… we find it also really useful for people that have low literacy levels for whatever reason… people whose first language is not English… people that are just short on time, people who are autistic or have other sensory processing issues (ORG_11).

The findings here—as well those above about people's preferences for different reading‐levels based on how they use the information—challenge the assumption of the cross‐group use or universality of ‘easy’ information.

## 4 Discussion

This paper has sought to understand decision‐making about the features of ‘easy’ information products in Australia, illustrating variation between provider organisations' choices on key features such as reading‐level and image selection, and uncovering the rationales for the feature‐decisions provider organisations make. The sections below summarise the findings, connection to existing literature, implications and limitations of the research.

### 4.1 Summary of Findings

The paper's findings formalise a lack of clarity in what features are termed Easy Read, Easy‐to‐Read, Easy English, Plain Language and Plain English in Australian ‘easy’ information production. Some organisations see clear differences between formats, others are not clear on the differences, and still others see, for example, Easy Read and Easy English as interchangeable terms. Despite the difficulties of consistent naming, there are however clearly different choices that ‘easy’ information provider organisations make: the reading‐level of ‘easy’ information ranges from Grade 1–2 to 6–9, while the images used vary between photos and graphics.

Perhaps the most important finding is however that ‘easy’ information provider organisations' choices about reading‐level and image selection reflect much about who their envisioned audience is. They make feature choices based *how* they see their envisioned audience *using* the information and about *what* will make their envisioned audience feel *recognised*/*empowered*.

### 4.2 Extension of Literature on Imagined ‘Local Audiences’

The findings here about reading‐level and image selection reflecting ‘easy’ information providers' envisioned audiences echo Chinn's (2019b) similar finding from the UK about ‘easy’ information providers meeting the preferred design elements of their own imagined ‘local audiences’; this is also essentially what these Australian ‘easy’ information provider organisations are doing.

The detail provided in this paper however helps to add more clarity on the rationale behind Chinn's ‘local audiences’ (2019b) issue: the ‘easy’ information providers in this paper demonstrate that they deeply understand the needs of the information users/community served by their specific organisation (i.e., their ‘local audience’), not only in *function* (i.e., logistic use of information) but also in *emotional experience* (i.e., how information makes them feel).

This is an important addition. The extra context it offers helps to demonstrate that the ‘local audiences’ that Chinn (2019b) describes are perhaps not served discretely due to unduly restricted/narrow practice by individual ‘easy’ information providers, but rather because each ‘easy’ information provider deeply knows their own users/community and aims to serve them as authentically as possible to what they say they need. This is commendable for taking a personalised approach to inclusive practice—yet with the caveat that, at least in Australia, there is need for ‘easy’ information provider organisations to better understand the uniqueness of each organisation's product for a particular audience and to step back from a tendency to assume that one product can meet the needs of everyone. While this is challenging in terms of a tendency to want to meet the principles of universal design, it reflects the reality of what is required to really meet the needs of specific audiences. In this way, the findings here help to extend Chinn's (2019b) seminal work in this field.

### 4.3 Practice Implications

One of the key practice implications of the paper's findings is that no single ‘easy’ information product will suit everyone—and that provider organisations need to more commonly recognise this. Set against literature questioning the efficacy of ‘easy’ information (Hurtado, Jones, and Burniston 2014; Sutherland and Isherwood 2016; Chinn and Homeyard 2017; Al‐Shirah 2023) and the support required to use it (Mander 2016; Chinn 2019a), this finding suggests the need for multiple audiences and specificity of audience to be greater considerations when judging the success of the variety of available ‘easy’ information products.

In practice, this may mean that ‘easy’ information provider organisations move towards clearly stating their intended/envisioned audience and the associated features/specifications of their documents upfront in their marketing and ‘easy’ materials. This might mean, for example, clearly stating whether a document is built to the specifications most commonly preferred by people with intellectual disability *or* people who use graphic‐based AAC systems *or* people who have low literacy but not necessarily a disability. This does not mean that other groups would be excluded from using the information, but rather that there is simply more clarity about who the information is primarily intended for.

If all ‘easy’ information provider organisations moved as a sector towards clearly stating audience upfront, it might also help to grow the Australian ‘easy’ information sector. One of the challenges of this sector is that it is common for ‘easy’ information work to be contracted to the same large providers time and again (Meltzer et al. 2024a), and, as a result, for particular ‘easy’ information products to be made to only one set of specifications. If it became common practice to state the precise audience/specifications of ‘easy’ information documents upfront, this could help to make the case to funders/commissioners of ‘easy’ information (e.g., government, private sector) about why multiple ‘easy’ materials with slightly different features are required. While obviously still operating within the limits of confined resourcing, making this point clearly could help assist the growth of the ‘easy’ information sector by clarifying the purpose of having multiple similar products.

Particularly in the Australian context, the other remaining challenge is the lack of formalised national guidance across a variety of ‘easy’ information products. It would be easy for this paper to recommend that all ‘easy’ information in Australia be consistently named according to set features—for example, ensuring Easy Read, Easy English and Plain Language documents each have delineated/agreed features via national guidelines. The reality is however that it would likely be difficult to gain universal agreement on such a delineation, given the extent to which—as noted earlier—the provider organisations involved are each so dedicated to meeting the needs of their particular users/community. While such guidance remains worthwhile, the requirement to be clear on intended audience and the associated specifications a document is perhaps a more realistic intermediate step.

### 4.4 Limitations

This paper has limitations. While the ‘easy’ information sector is small, the sample is still comparatively small—only 12 organisations. A larger sample would yield more insights.

Further, in any research touching on disability, it is critical to centralise the perspectives of people with disability. As this was organisation‐focused research, the perspectives of people with disability were only included where participating staff of ‘easy’ information provider organisations had a disability. This is a significant limitation, as the views of people with intellectual disability should systematically underpin all research relevant to them. A more thorough study would craft, check and confirm the findings with people with intellectual disability directly.

Finally, ‘easy’ information production continues to progress; it is a limitation that this research does not address new developments, including the use of generative artificial intelligence in accessible information production. This is an area for future research.

### 4.5 Concluding Comments

This paper makes an important contribution to illustrating the rationales behind decision‐making in the Australian ‘easy’ information sector. This is useful for better understanding why different ‘easy’ information formats are made the way they are. Ultimately, Australian ‘easy’ information provider organisations each seek to meet the needs of the users/community that follow their products and to ensure that their products are not only useable by them but also make them feel recognised/empowered. This is a worthwhile contribution to the work of inclusion—the remaining task is in how to organise around the work of the provider organisations as a group, so that there is more clarity about all ‘easy’ information products available and whom they are intended to serve.

## Ethics Statement

The project received ethics approval from the University of New South Wales' Business School's Human Research Advisory Panel (HC230201). All participants and their organisations provided informed consent.

## Conflicts of Interest

Ariella Meltzer is a Board member of an organisation that makes ‘easy’ information.

## Data Availability

Research data are not shared.
